# Identification of a novel cancer stem cell subpopulation that promotes progression of human fatal renal cell carcinoma by single-cell RNA-seq analysis

**DOI:** 10.7150/ijbs.46645

**Published:** 2020-10-17

**Authors:** Xiu-wu Pan, Hao Zhang, Da Xu, Jia-xin Chen, Wen-jin Chen, Si-shun Gan, Fa-jun Qu, Chuan-min Chu, Jian-wei Cao, Ying-hui Fan, Xu Song, Jian-qing Ye, Wang Zhou, Xin-gang Cui

**Affiliations:** 1 Department of Urology, The Gongli Hospital of Second Military Medical University, Shanghai 200135, China; 2 Department of Urology, The Third Affiliated Hospital of Second Military Medical University, Shanghai 201805, China; 3 Department of Urology, The Changzheng Hospital of Second Military Medical University, Shanghai 200003, China; 4 Department of Bone Tumor Surgery, The Changzheng Hospital of Second Military Medical University, Shanghai 200003, China; 5 Department of Anesthesiology, Renji Hospital, School of Medicine, Shanghai Jiaotong University, Shanghai 200127, China; 6 Department of Urology, The Seventh People's Hospital of Shanghai University of Traditional Chinese Medicine, Shanghai 200137, China

**Keywords:** Collecting duct renal cell carcinoma, Single-cell RNA sequencing, Cancer stem cell, Cellular heterogeneity, Therapeutic strategy

## Abstract

**Background:** Cancer stem cells (CSCs) are biologically characterized by self-renewal, multi-directional differentiation and infinite proliferation, inducing anti-tumor drug resistance and metastasis. In the present study, we attempted to depict the baseline landscape of CSC-mediated biological properties, knowing that it is vital for tumor evolution, anti-tumor drug selection and drug resistance against fatal malignancy.

**Methods:** We performed single-cell RNA sequencing (scRNA-seq) analysis in 15208 cells from a pair of primary and metastatic sites of collecting duct renal cell carcinoma (CDRCC). Cell subpopulations were identified and characterized by t-SNE, RNA velocity, monocle and other computational methods. Statistical analysis of all single-cell sequencing data was performed in R and Python.

**Results:** A CSC population of 1068 cells was identified and characterized, showing excellent differentiation and self-renewal properties. These CSCs positioned as a center of the differentiation process and transformed into CDRCC primary and metastatic cells in spatial and temporal order, and played a pivotal role in promoting the bone destruction process with a positive feedback loop in the bone metastasis microenvironment. In addition, CSC-specific marker genes BIRC5, PTTG1, CENPF and CDKN3 were observed to be correlated with poor prognosis of CDRCC. Finally, we pinpointed that PARP, PIGF, HDAC2, and FGFR inhibitors for effectively targeting CSCs may be the potential therapeutic strategies for CDRCC.

**Conclusion:** The results of the present study may shed new light on the identification of CSCs, and help further understand the mechanism underlying drug resistance, differentiation and metastasis in human CDRCC.

## Introduction

Fatal malignancy is the most daunting challenge for clinicians due to lack of profound understanding and effective treatment of the disease 1, 2. Rapid progression of this malignant disease is the leading cause of devastating consequences including metastasis, recurrence and drug resistance. In the heterogeneous composition of tumor tissues, cancer stem cells (CSCs) are rare tumor cells with unique properties, playing a crucial role in functional assays of self-renewal, differentiation and tumor initiation 3-5. Therefore, questing on the separation, identification and properties of CSCs could provide very important information clues for anti-tumor drug selection and drug resistance 5, 6.

Collecting duct renal cell carcinoma (CDRCC) originating from the epithelium of the distal collecting duct is a subtype of renal cell carcinoma (RCC), accounting for about 2% of all RCCs 7, 8. Clinically, CDRCC is characterized as a highly aggressive disease with approximately 80% patients developing metastasis to the lymph nodes (LN), bone, lung and liver 7, 9. According to the Surveillance, Epidemiology and End Results (SEER) database analysis from 98 CDRCC patients, the 3-year CDRCC-related survival rate for localized, regional and distant disease was 93%, 45%, and 6% respectively, as a result of an extremely poor prognosis for metastatic CDRCC patients with median overall survival (OS) at less than 12 months 10. Except for exceptional cases, the response of most CDRCC patients to molecular targeted therapy, immunotherapy, chemotherapy or even their combination is usually poor 11, 12.

Cellular heterogeneity across primary and metastatic RCC dramatically affects the sensitivity of anti-tumor therapy owing to incomplete response to all tumor cell subpopulations 13, 14. To reveal the intercellular heterogeneity leading to CDRCC progression in an unbiased manner, we used scRNA-seq to explore the relationship between the complex tumor cellular ecosystem and clinical biological evolution using detailed computational analysis.

## Results

### Identification of human CDRCC cell populations

**Figure 1:**
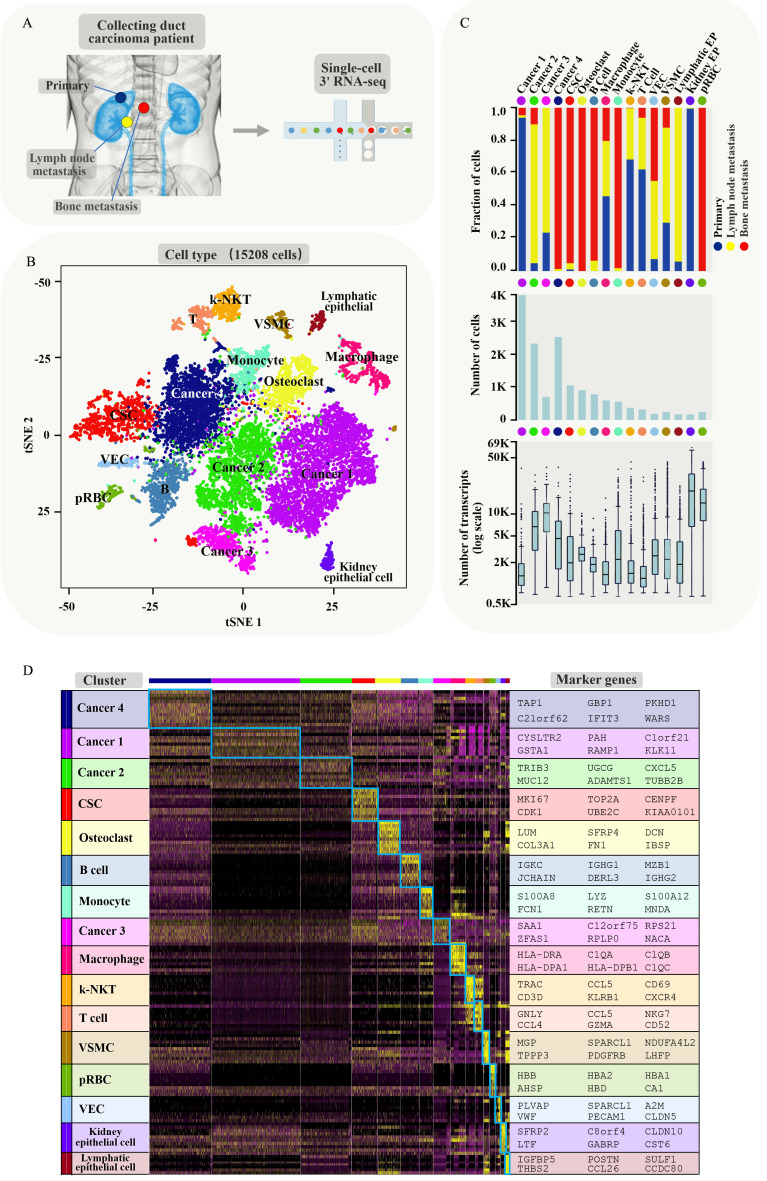
** Brief description of 15208 single cells from primary tumor and metastatic lesions of CDRCC.** A, Overview of the sample source: Three samples of the primary tumor, LN metastasis, and bone metastasis of CDRCC were collected from the same patient simultaneously, followed by single-cell RNA sequencing protocol. B, The t-SNE plot shows that 15208 cells were divided into 16 cell clusters, including four cancer clusters, one cancer stem cell cluster (CSC), two epithelial cell clusters (Kidney epithelial cells and Lymphatic epithelial cells), five immune cell clusters (B cells, T cells, kidney-natural killer T cells (k-NKT), monocyte and macrophage), one Vascular endothelial cells (VEC), one osteoclast (OC) cluster, one vascular smooth muscle cell (VSMC) cluster, and one progenitor red blood cell (pRBC) cluster. We labeled them in different colors and one dot represents one single cell. C, Three graphs (from top to bottom) show the fraction of cells (blue bar: primary tumor, red bar: bone metastasis, yellow bar: LN metastasis) , the cell number, and the number of transcripts in 16 cell clusters. D, Cluster heat map display: the cells are subdivided into 16 clusters (represented by colored bars at the top) and defined by the marker genes. The classification of clusters is displayed on the y-axis (left) and the corresponding marker genes are displayed on the y-axis (right).

In order to understand intercellular heterogeneity of human CDRCC, we collected tumor tissues simultaneously from the same patient who underwent cytoreductive nephrectomy and spinal biopsy (Supplementary Fig. S1), and performed tumor tissue digestion into single cells, quality filtering and sequencing (Fig. 1A). Finally, we acquired 15,208 malignant, immune and stromal cells isolated from CDRCC primary and metastatic tumor tissues, including 4984 primary tumor cells, 3568 LN metastatic tumor cells, and 6656 bone metastatic tumor cells.

We next used nonlinear dimensionality reduction (t-distributed stochastic neighbour embedding, t-SNE) 15 and graph_based Louvain clustering algorithm 16 to investigate cell distribution and heterogeneity of CDRCC in accordance with tumor tissue origins (Supplementary Fig. S2A). All the tumor cells were annotated as 16 clusters through unsupervised clustering with 4052 signature genes (Supplementary Table 1). Cell clusters were annotated as four cancer clusters (cancer 1 mainly derived from primary tumor, cancer 2 and 3 mainly derived from LN metastasis and cancer 4 mainly derived from bone metastasis), one cancer stem cell cluster (CSC), two epithelial cell clusters (Kidney epithelial cells and Lymphatic epithelial cells), five immune cell clusters (B cells, T cells, Kidney-natural killer T cells (k-NKT), Monocyte and Macrophage), one vascular endothelial cell(VEC) cluster, one osteoclast (OC) cluster, one vascular smooth muscle cell (VSMC) cluster, and one progenitor red blood cell (pRBC) cluster (Fig. 1B and Supplementary Fig. S2B,C). Among the four cancer clusters, cancer 1 mainly derived from the primary tumor, cancer 2 and 3 mainly derived from LN metastasis and cancer 4 mainly derived from bone metastasis (Fig. 1C). Significant differences in transcriptional activity and signature gene expression were revealed between the different clusters (Fig. 1C,D).

### Differentiation of intercellular heterogeneity in malignant and nonmalignant subpopulations

To investigate genetic heterogeneity between malignant and nonmalignant cells, we chose four cancer clusters (cancer 1-4) and CSC cluster as malignant cell subpopulations and four normal cell clusters (three epithelial cell clusters and a VSMC cluster) as the control to infer the large-scale copy number variations (inferCNVs). The inferCNVs in malignant cells showed extensive chromosomal losses in 1p, 3p, 4q, 9 and 11. In addition, extensive chromosomal gains were observed in 1q, 12 and 20 (Supplementary Fig. S3A,B; the gene list of inferring contingent negative variation (interCNV) is shown in Supplementary Plot 1), which is highly consistent with the previous reports 17. Interestingly, significant CNV loss on chromosome 6 was observed in Cancer 2 and 3 (LN metastasis cells), and significant CNV gain was observed on chromosome 17 in Cancer 1 (primary cells). Several studies have demonstrated that chromosomal losses in 9p and gains in 20p are closely associated with poor survival of RCC patients 18, 19.

### Identification and characterization of CSC population

**Figure 2:**
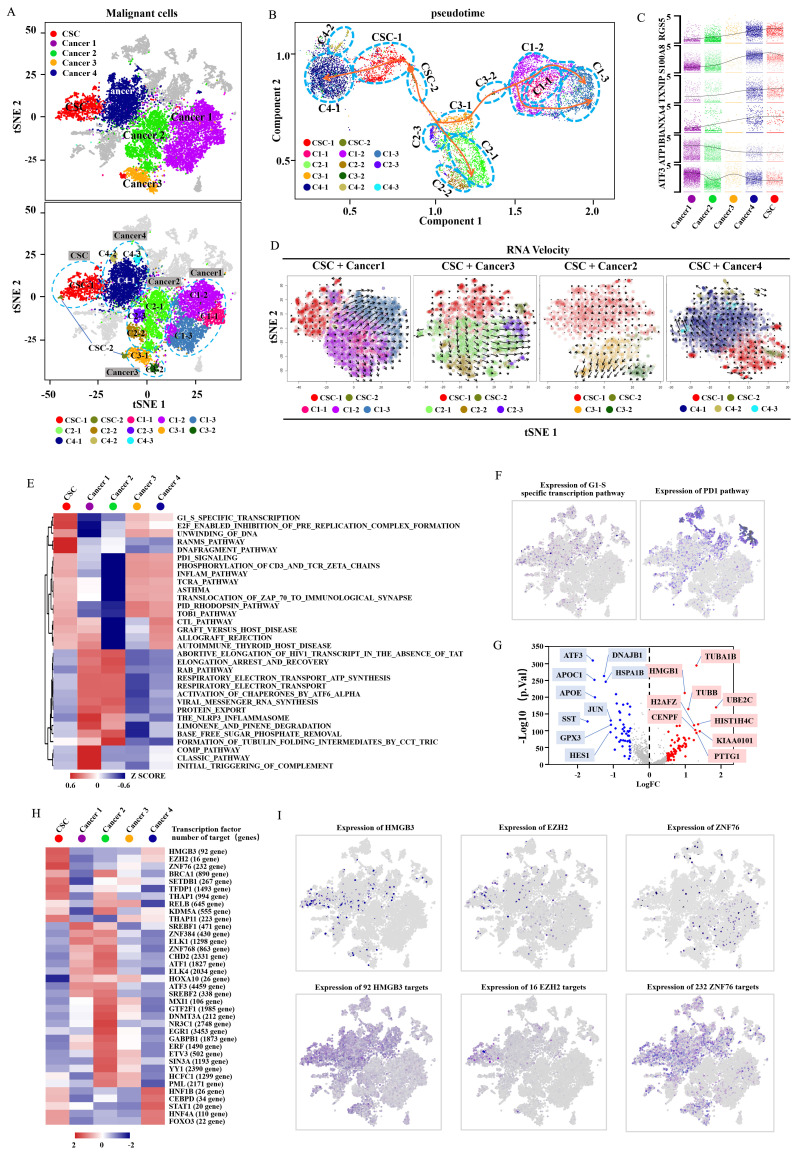
** Identification and characterization of malignant cell populations. A**, The t-SNE plot shows several clusters of malignant cell populations including CSC and Caner1-4, and the five types of cluster are respectively divided into several subclusters (CSC: CSC-1, CSC-2; Cancer1: C1-1, C1-2, C1-3; Cancer2: C2-1, C2-2, C2-3; Cancer3: C3-1, C3-2; Cancer4: C4-1, C4-2, C4-3).** B**, A pseudotemporal ordering for the similarity of cancer subpopulations with developmental lineages using an improved pseudotime trajectory axis with a branched structure. The arrows represent the differentiation directions and dots in the different circles with dotted line represent the cancer subclusters.** C**, The dynamic expression of the key regulator genes (RGS5, S100A8, TXNIP, ANXA4, ATP1B1, ATF3) according to (**B**) in tumor differentiation among five cell clusters (Cancer1-4,CSC).** D**, RNA Velocity analysis illustrates the extent and direction of malignant cell differentiation in the t-SNE plot between the CSC and cancer1-4 clusters. The direction of arrow represents the future state of the cells.** E**, The heat map demonstrates the enrichment of top 32 pathway activities in malignant clusters (CSC, Cancer1-4) via GSVA (P<0.01, see methods).** F**,t-SNE plot showscell enrichment in G1/S-specific transcription and pd-1 pathway.** G**,The volcano plot explains the differentially expressed genes between the CSC and cancer cluster, where red dots represent the genes expressed strongly in the CSC cluster, and blue dots represent the genes expressed strongly in the cancer cluster(P<0.001).** H**, The heat map exhibits the average regulon activities of transcription factors between CSC cluster and cancer clusters(top 10 regulons in each cluster were selected ). The number in the parenthesis shows the downstream genes regulated by the corresponding transcription factors. **I**, The t-SNE plot(top) shows the top three highly expressed transcription factors in CSC, and the t-SNE plot(down) shows the corresponding downstream target genes.

To further reveal the relationship between cancer cells and CSCs, we subdivided malignant cells into subclusters and identified each subcluster by cell-specific marker genes (Fig. 2A, Supplementary Fig. S4A-C). The well-known clear cell renal cell carcinoma (CCRCC) CSC markers (ALDH1A1, CD44, CXCR4, DCLK1, CD105, ITGB1, NT5E, PIK3R1, PROM1 and THY1)^20^-^27^ were displayed in the featurePlot to verify the expression in the identified CDRCCCSC cluster (Supplementary Fig S5A, B). It was found that ALDH1A1, DCLK1, ITGB1 and PIK3R1 were also expressed in the identified CDRCC CSC cluster. However, these CCRCC CSC marker genes were not specifically expressed genes. We attributed the differences to the different origination from kidney epithelial cells between CCRCC and CDRCC.

To investigate the potential evolution among CDRCC cell populations, we used the Monocle method to reveal a pseudotemporal ordering for the similarity of tumor subclusters with developmental lineages. We applied the improved pseudotime trajectory axis with a tree-like structure, and found that CSCs positioned as a center of the differentiation process sequentially transformed into cancer 1-4 clusters. Therefore, we acquired three trajectory axes in an unbiased manner, including CSC to Cancer 1/3, Cancer 2 and Cancer 4, and specific representative genes that marked the differentiation process (Fig. 2B,C and Supplementary Fig. S6A,B). In addition, we used t-SNE with RNA velocity analysis as a visual and intuitive way to observe the extent and direction of tumor cell differentiation through the direction of the arrows in the figure (Fig. 2D and Supplementary Fig. S6C). Cells in the CSC cluster showed the highest degree of malignancy and were regarded as a starting point to each direction of differentiation. These results suggest that CSCs have the capacity of multi-directional differentiation to maintain the vitality of each malignant cell cluster.

To further define the distinguishing functions of these malignant subpopulations, we used Gene Set Variation Analysis (GSVA) and Gene Set enrichment analysis (GSEA) to compare signaling pathway activities and found significant phenotypic diversity between these malignant subpopulations. It was found that the CSC cluster showed a highly enriched signature in G1/S specific transcription, Ranms signaling pathway, E2F enabled inhibition of pre replication complex formation, DNA fragment pathway, cell cycle, DNA replication and spliceosome, which are closely associated with cell proliferation, DNA replication, DNA stability and transcriptional regulation, suggesting that CSCs possess a strong ability of active self-renewal (Fig. 2E,F, Supplementary Fig. S7 and Supplementary Table 2-4). It is worth mentioning that the PD-1 signaling pathway was also enriched in CSC cluster, indicating that immunological checkpoint inhibitor (anti-PD-1/PD-L1) may represent a potential therapeutic strategy for CDRCC (Fig. 2E,F).

Compared with the cancer clusters, the CSC cluster was highly differentially expressed with genes HMGB1, H2AFZ, KIAA0101 at *p*-value <1e-10, which was verified by CSC-related specific genes in previous studies (Fig. 2G, Supplementary Fig. S8 and Supplementary Table 5). In addition, single-cell regulatory network inference and clustering (SCENIC) was applied to further assess potential expression differences in transcription factors between the CSC and cancer clusters 28. Compared with the cancer clusters, HMGB3, EZH2 and ZNF76were identified as candidate transcription factors whose expressions were specifically regulated in the CSC cluster (Fig. 2H,I). It is noteworthy that EZH2 was found to be a histone methyltransferase that regulated self-renewal of CSCs to promote the metastasis of cancer cells by epigenetic silencing of target genes 29, 30.

Subsequently, we analyzed the key molecules for current targeted therapies to assess potential therapeutic responses for human CDRCC. Sunitinib, Pazopanib and Sorafenib are multi-target receptor tyrosine kinase inhibitors widely used for RCC. We found that target molecules of these inhibitors, including VEGFR1, VEGFR2, VEGFR3, VEGFA and PDGFRB, were markedly expressed only in cancer 1 and 2 clusters (primary tumor and LN metastasis), while their expressions in cancer 4 (bone metastasis) and CSC clusters were relatively low (Supplementary Fig. S9A). These results imply that CDRCC bone metastatic cells may be associated with intrinsic resistance to multi-target receptor tyrosine kinase inhibitors, which may explain why this patient with bone metastasis was insensitive to pazopanib after cytoreductive nephrectomy. PARP1, PIGF, HDAC2, and FGFR3 were highly expressed in malignant cell clusters, especially in the CSC cluster, which was further verified by the immunofluorescence double labeling method (Supplementary Fig. S9A,B). Therefore, inhibitors currently available for these genes, including PAPR inhibitor (Niraparib), PIGF inhibitor (ziv-aflibercept), HDAC2 inhibitor (Belinostat, Romidepsin, Vorinostatt) and FGFR inhibitor (Derazantinib, AZD4547, BGJ398, Dovitinib), may be the potential therapeutic strategies for CDRCC. As previously reported, targeting CSCs could effectively inhibit the source of differentiated progeny of malignancies and improve the sensitivity of anti-tumor treatment 31.

### Identification of the potential evolution process in the bone metastasis microenvironment

**Figure 3:**
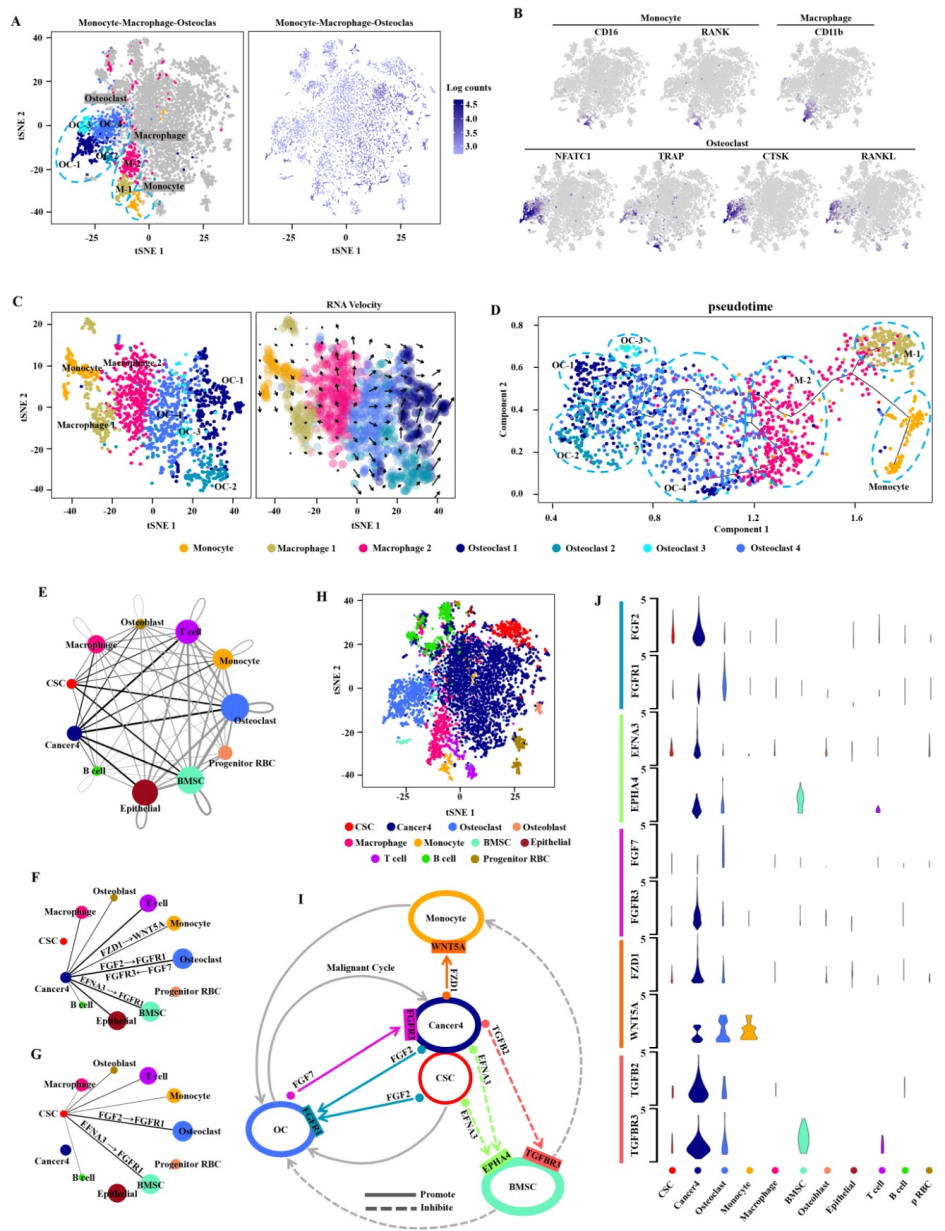
** Cell potential evolution and cell-cell communication in the bone metastasis microenvironment. A**, t-SNE plot shows the monocyte, macrophage and osteoclast clusters which labeled with relevant colors and the transcripts(UMI) detected in all cells in the bone metastasis.** B**, t-SNE plot shows the Monocyte-associated marker genes (CD16, and RANK), macrophage-associated marker gene(CD11b), and osteoclast-associated marker genes (NFATC1, TRAP, CTSK and RANKL) respectively.** C**, RNA velocity analysis demonstrates the differentiation direction of the neighboring clusters among the bone metastasis related cell clusters. The arrows represent the direction of the differentiation inferred by the average velocity.** D**, Analysis of the improved pseudotime trajectory axis shows that adjacent clusters along the developmental trajectory exhibit a monocyte-macrophage-osteoclast axis.** E**, The network diagram (drawn by cytoscape version 3.7.1) shows the communication between the 11 cell clusters, each circle representing one cell cluster. The thickness of the line represents the strength of the interaction between the two cell clusters and the size of circle represents the strength of the interaction between the corresponding cell cluster and the other cell clusters. The lines across itself represent the autocrine and the thicker lines and bigger circles indicate the stronger interactions. The sc-RNA seq data were analyzed by CellPhoneDB(see methods). **F**,**G**, Communication between Cancer4 cluster and other cell types(**F**) and communication between CSC cluster and other cell types(**G**).** H**, t-SNE plot shows the 11 cell clusters from the bone metastasis sample.** I**, The circular diagram shows the cluster of malignant cells (including CSC cluster and Cancer4) interacting with the bone related cells (including monocyte, BMSC and OC) and their communication with each other in the bone metastasis microenvironment. The solid lines with arrow represent the promotion and the dotted lines with arrow represent the inhibition. **J**, The violin plot reveals the expression of important ligand-receptor-pairs of the circular diagram (**i**) among the bone metastasis cell clusters.

It is common knowledge that distant metastasis occurs at terminal stage during the clinical biological evolution of various cancers, and is intimately related to the survival of most cancer patients 32, 33. To clarify the cell composition in the bone metastatic tissues, we validated these cells with the monocyte-related markers CD16 and RANK, the macrophage-related marker CD11b, and the osteoclast-related markers NFATC1, TRAP and CTSK as the monocyte cluster, macrophage cluster, and osteoclast cluster respectively by using t-SNE analysis (Fig. 3A, B). The evolution-based trajectory was supported by improved pseudotemporal analysis and RNA velocity analysis, showing that the neighboring clusters along developmental trajectory presented a monocyte-macrophage-osteoclast axis, which is fully consistent with the previous report 34 (Fig. 3C, D).

Bone metastasis is a key prognostic factor affecting survival in RCC patients 35. To characterize the microenvironment of bone metastasis in CDRCC, we applied CellPhoneDB to reveal the cell-cell communication between malignant and bone related cells with ligand-receptor complex (Fig. 3E-H). We found that cells of cancer 4 cluster had high-level expression of FZD1 which interacted with the receptor WNT5A of monocytes (Fig. 3I, J), and the ligand-receptor complex of FZD1/WNT5A played a key role in cancer cells by promoting the proliferation and differentiation of monocytes into osteoclasts 36 (Fig. 3I, J). TGFB2 and EFNA3 were highly expressed in malignant cells, and their receptors (TGFBR3 and EPHA4) were specifically expressed in bone mesenchymal stem cells (BMSCs) (Fig. 3I, J). The ligand-receptor complexes of TGFB2/TGFBR3 37 and EFNA3/EPHA4 38 inhibited the proliferation of BMSCs, thus reducing the inhibitory effect of BMSCs on osteoclasts and monocytes. FGF2, which was highly expressed in both cancer 4 cells and CSCs, interacted with the receptor FGFR1 in osteoclasts. Meanwhile, FGF7 secreted from osteoclasts affected the receptor FGFR3 in cancer 4 cells. FGF2/FGFR1 39 and FGF7/FGFR3 40 ligand-receptor complexes formed a positive feedback loop (malignant cycle) between bone metastatic malignant cells and the monocyte-osteoclast system. These findings reveal the mechanism of bone destruction promoted by tumor cells in the bone microenvironment. Therefore, our results may provide a novel treatment strategy for advanced metastatic CDRCC with application of FGF/FGFR inhibitors, such as Derazantinib, AZD4547, BGJ398 and Dovitinib 41-44.

### Excellent clinical prognostic abilities of malignant subpopulations associated genes identified by scRNA-seq analysis for human CDRCC

**Figure 4:**
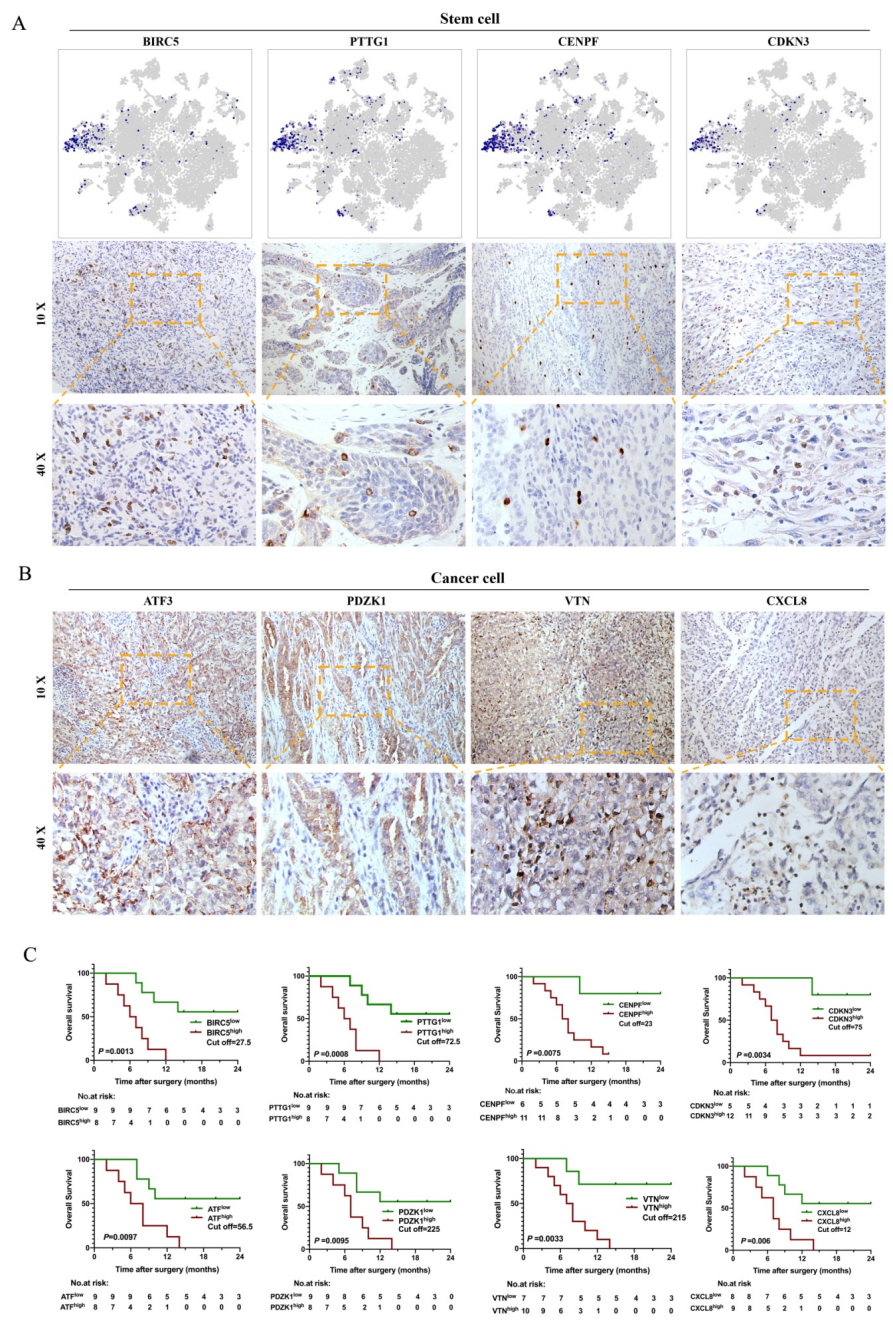
** Immunohistochemistry and survival analysis of CDRCC. A**, t-NSE plot shows the expression distribution of BIRC5, PTTG1, CENPF and CDKN3 in 15208 cells and the corresponding immunohistochemical images using the 10× and 40× objective. **B**, IHC images show the expression of Cancer cluster-associated genes ATF3, PDZK1, VTN, and CXCL8 in CDRCC tissues. **C**, Kaplan-Meier survival curves for CDRCC patients show the relationship between gene expression level in (**A**) and (**B**) and survival prognosis (n=17 ,p<0.01).

Our study showed that malignant subpopulations were relatively independent and connected to each other, especially for the CSC cluster, which played a key role in the differentiation of tumor cells and impacted the microenvironment of bone metastasis in human CDRCC. Therefore, we assumed that CSC-associated genes might be able to provide important prognostic information. To test this, we assessed the prognostic value of scRNA-seq derived gene signature from 17 tissue samples, which were identified as CDRCC by three experienced pathologists independently (Supplementary Table 6). It was found that CSCs specifically expressed BIRC5, PTTG1, CENPF and CDKN3 genes and presented a scattering distribution in the bulk tumor tissues, which further verified the relatively rare characteristics of CSCs (Fig. 4A). In addition, high expression of BIRC5, PTTG1, CENPF and CDKN3 was significantly associated with poor prognosis, as represented by the fact that most patients died within 12 months (*P*<0.01) (Fig. 4C). As expected, cancer cluster-related genes ATF3, PDZK1, VTN and CXCL8 were highly expressed in CDRCC tissues, and patients with high expression of these genes had significantly poor prognosis (*P*<0.01) (Fig. 4B, C). These data suggest that the malignant subpopulation-associated genes that we identified by scRNA-seq in the present study possessed a superior clinical prognostic value.

## Discussion

Intercellular heterogeneity of tumors in different lesions of the same patient includes intertumoral heterogeneity (different lesions, such as primary and metastatic sites) and intratumoral heterogeneity (difference within the same lesion), which are composed of a variety of different tumor cell subpopulations.

Different tumor cell subpopulations present different biological phenotypes, such as immune response, malignant proliferation, invasion and metastasis, which ultimately lead to differences in sensitivity to antitumor agents, radiotherapy and chemotherapy 45. As a new technology, scRNA-seqmakes it possible to uncover genetic and transcriptional features of thousands of single tumor cells to identify the cellular components, endogenous heterogeneity and biological evolution of tumors, which provides a reliable manner to predict tumor response to anticancer agents for refractory cancers 46. How to eliminate inter-individual heterogeneity is especially critical during demystifying biological properties of CSC population in scRNA-seq study. However, most of these studies lacked homologous specimens, because of difficulties in obtaining a pair of primary and metastatic samples from the same patient during the same period. Our study provides the accuracy of bioinformatics analysis by effectively evading the inter-individual heterogeneity.

We used scRNA-seq and immunohistochemical analysis to identify the CSC population of CDRCC and characterize the properties of CSC subpopulation. Compared with other cell subpopulations, CSCs were relatively smaller in quantity, but possess an active self-renewal capacity through upregulation of cell mitosis-related signaling pathways, suggesting that these cells have a high proliferating ability. Meanwhile, SCENIC analysis showed that EZH2 was an important candidate transcription factor that was highly expressed in CSC subpopulation to specifically regulate CSC-related gene expression and promoting self-renewal of CSCs, finally leading to cancer cell metastasis 29, 30. In addition, through a visual and intuitive way including Monocle and t-SNE + RNA velocity analysis, we showed three unsupervised differentiation trajectories of CSCs to primary tumor cells (CSC to Cancer 1/3), LN metastasis tumor cells (CSC to Cancer 2) and bone metastasis tumor cells (CSC to Cancer 4), suggesting a superior ability of multi-directional differentiation. The tumor metastasis ability is an inherent characteristic of the CSC subpopulation, known as metastatic CSCs (mCSCs) 47. mCSCs as cell origin can not only differentiate into metastatic tumor cells, but regulate the local microenvironment to promote metastasis^47^. Our study showed that the proportion of mCSCs accounts for most of all CSCs, especially for bone mCSCs. Bone mCSCs of CDRCC had the active features of self-renewal and differentiate into cancer cells. In addition, bone mCSCs of CDRCC promoted the progress of bone metastasis by regulating the bone microenvironment. These findings reveal that CSCs of CDRCC have a superior metastatic activity.

Bone metastasis is a common complication of advanced RCC, and an important signal for poor prognosis and even death in patients with malignant tumors 48. Like the “seed and soil” hypothesis, interplay between specific cancer cells and the suitable bone microenvironment promotes the unbridled growth of cancer cells. Osteolytic destruction is the main characterization of RCC bone metastasis, a process that is caused by osteoclast stimulation rather than by the direct effect of cancer cells on bones 48, 49. Therefore, the active state of osteoclasts affects the fate of RCC bone metastasis. According to the cell-cell communication between malignant cells and bone related cells with ligand-receptor complex, we found that CDRCC cells+CSCs increased the osteoclast activity through multiple pathways (Fig. 3i): (1) the ligand-receptor complex of FZD1/WNT5A between cancer cells and monocytes promoted the proliferation and differentiation of monocytes into osteoclasts; (2) the ligand-receptor complexes of TGFB2/TGFBR3 and EFNA3/EPHA4 between cancer cells+CSCs and BMSCs inhibited the proliferation of BMSCs to reduce the inhibitory effect of BMSCs on osteoclasts and monocytes; (3) the ligand-receptor complexes of FGF2/FGFR1 and FGF7/FGFR3 formed a positive feedback loop (malignant cycle) between cancer cells+CSCs and osteoclasts. These findings provide a key compelling strategy for the treatment of CDRCC through inhibiting the interaction of cancer cells and the bone microenvironment.

Surgery is a curative treatment option for early localized CDRCC 17. However, most CDRCC patients had presented distant metastasis at an initial visit 7. Patients with metastatic CDRCC show limited response to chemotherapy, radiotherapy and other conventional treatments. In the era of targeted therapy, some studies 12, 51 have demonstrated that targeted therapeutic agents (sunitinib or sorafenib) confer survival benefits in exceptional CDRCC patients. VEGFR-targeted therapy for CDRCC patients is inferior compared with that in patients with other non-clear cell RCC 51. According to the molecular features of CDRCC in our study, PARP, PIGF, HDAC and FGFR inhibitors for effectively targeting CSCs may be the potential therapeutic strategies for human CDRCC. Among them, FGFR inhibitors in particular can block the cell-cell communication between cancer cells+CSCs and osteoclasts in the bone microenvironment, suggesting that FGFR inhibitors may inhibit the progress of CDRCC bone metastasis. These findings provide valuable clues for targeting CSCs to block the source of differentiated progeny of CDRCC tumor cells, which can overcome such highly fatal diseases from the root cause.

Although our research is based on the in-depth characterization of CDRCC molecular features by scRNA-seq analysis, it still has some limitations due to the single patient with a pair of primary and metastatic samples. In addition, it cannot eliminate the bias of analysis results from a single database. As CDRCC is a rare and fatal subtype of RCC, there is a low-probability to collect more fresh CDRCC tissues to carry out scRNA-seq analysis. To minimize this limitation, we collected 17 CDRCC tissue samples from previous surgeries to assess the prognostic value of novel genes identified by scRNA-seq analysis. In addition, it is for the first time that we explored the complex tumor cellular ecosystem and biological evolution of CDRCC using an unbiased scRNA-seq analysis, which may provide a basis for understanding the biological behavior of CDRCC, though more cases and pathological studies are needed to confirm our findings in the future.

In summary, our study has identified a novel CSC subpopulation, which may signify important progress in understanding the spatial and temporal order in clinical biological evolution of human CDRCC. This groundbreaking discovery may provide a new treatment strategy through targeting CSC related genes and enrich our understanding about the interaction between the bone microenvironment and cancer cells. Our study also highlights that malignant-specific genes identified by scRNA-seq are closely related to clinical prognosis of CDRCC. This visual presenting method for multi-dimensional characterization of human CDRCC could also be applied to other fatal cancers.

## Methods

### Preparation of single-cell suspensions

A female patient diagnosed with distant metastatic RCC (thoraco spin, lumbar spine and retroperitoneal lymph nodes) by CT, MRI and PET-CT scanning was enrolled in this study for single-cell RNA-seq analysis. Cytoreductive nephrectomy and spinal biopsy were carried out to collect fresh tissue specimens (primary tumor, LN metastasis and lumbar spine metastasis), which were pathologically confirmed as CDRCC. In addition, we collected 17 CDRCC tissue samples which were previously obtained by surgical resection or tissue biopsy of renal lesions from February 2009 to February 2018. The histology of all patients were diagnosed with CDRCC by IHC analysis (AE1/AE3, Ki 67, CK19, CK 7, PAX 8, P504S, Ini, 34βE12, Vimentin, CK7, CD10, P63, GATA3 and EBER) of three experienced pathologists. The clinical features of these patients were summarized in Supplementary Table 6. This study was approved by the ethics committee of Gongli Hospital (Shanghai, China; No.2018-012). All patients in this study provided their informed consent.

Three samples removed from the patient in the operating room were separately rinsed with normal saline to eliminate the influence of RBC and preserved in 4℃ liquid medium, then transported immediately to the laboratory. Upon arrival, the samples were rinsed with phosphate buffer saline (PBS) solution again and minced on ice into tiny cell clumps < 1mm^3^ in volume. Each sample was then transferred into a 15ml centrifuge tube with 10ml digestion medium with 0.75 mg/ml collagenase I (Sigma), 2mg/ml collagenase Ⅳ (Sigma), 0.0025 mg/ml DNase Ⅳ (Sigma), and 0.2mg/ml hyaluronidase I-S (Sigma) in 0.25% Trypsin (ThermoFisher Scientific). The specimens were incubated in a 37 °C thermostatic shaker with manual mix every 10 min until the cell clumps were digested to single cells. Next, the samples were filtered using 40-μm nylon meshes (Corning) to remove cell debris and clusters. After centrifugation at 300×g and 4°C for 5 min, the cell pellet was re-suspended in 2mL red blood lysis buffer and incubated for 5min at room temperature. Cell suspension was centrifugated under the same condition and the pellet was re-suspended in 1mL PBS solution. Automatic cytometry (Luna) was used to determine the cell concentration and the sample volume was calculated based on the optimal cell sampling concentration supplied by 10x Genomics official website (https://www.10xgenomics.com/) and the target capture number. If the calculated concentration was too high, the liquid volume would be adjusted to the appropriate concentration and counting was performed again. Once the desired cell suspension was obtained, the sample was immediately placed on ice for subsequent GEMs preparation and reverse transcription.

### Droplet-based scRNA-seq

The prepared single-cell suspensions were barcoded to scRNA-seq libraries with the Chromium Single Cell 3' Library, Gel Bead & Multiplex Kit and Chip Kit (10x Genomics). About 16516 cells were totally captured at last in all the samples. The V2 barcoding chemistry of 10x Genomics was applied to treat the cell RNA. Each sample was plated in the same cell in the special 10x plate, ensuring that each cell was treated with the same mixture in the same cellular channel. All samples were processed synchronously in the same thermal circulator. Libraries were sequenced on an Illumina Hiseq X, and labeled in the human genome (build Grch38) under the operation of CellRanger (10x Genomics). The gene location was annotated with Ensembl build 95.

### Quantification of single-cell gene expression and classification of principal cell types

Single-cell expression matrices for CCRCC CSCs were obtained from the Gene Expression Omnibus (GEO; https://www.ncbi.nlm.nih.gov/, GSE110680) 52. The data were processed in the same way as CDRCC. Original sequencing data matrices from CellRanger (version 3.0.2) were imported to R (version 3.5.2-Eggshell Igloo), and integrated with Seurat R package (version 2.3.4) 53. To guarantee the quality of sequencing, the cells with <200 or > 5000 genes were depleted from the original data. Dimensionality reduction was performed in the remaining 15208 cells for a subset of highly variable genes in the gene expression matrices. With the help of FindVariableGenes function of Seurat package, we selected the highly variable genes by calculating variance to mean expression ratios in evenly sized groups. Genes with a mean expression between 0.05 and 10, and dispersion > 0.5 were selected. Cellular read count, sample source and mitochondrial mRNA percentage were linearly regressed against all genes under the guideline of Seurat's ScaleData function. Principal component analysis (PCA) was then conducted and top 30 principal components were used for clustering with the Louvain graph-clustering method 16. The clusters were projected on the t-SNE plot 15. As to subclusters, the same methods were used for recognizing the variable genes, reducing dimensions and clustering.

### Identification of marker genes

To identify marker genes of these 16 clusters, we contrasted cells from one cluster to all other cells using the Seurat *FindAllMarkers* function. The marker genes had to express in more than 10% cells in its cluster and the average expression in corresponding cluster was required 0.25 log2 fold changes higher than that in other clusters. Among the 16 clusters, 5 clusters (Cancer 1-4 and CSC clusters) were further divided into 13 subclusters. The marker genes of 13 subclusters were recalculated.

### Correlation to clinical data

To validate the results of scRNA-seq analysis, we selected totally 8 highly expressed genes in CSC cluster (n=4) and Cancer cell clusters (n=4). By immunohistochemistry (IHC), we stained sections of 5-µM thickness from the paraffin blocks of 17 CDRCC patients (Supplementary Table 6). According to the immunohistochemical scores, Kaplan-Meier curve was drawn to present the relationship between the expression level and survival time. Second, to verify the possible therapy drugs to CDRCC, we selected 1 CSC-related gene and 4 targeted therapy genes to carry out double immunofluorescence labeling staining to detect the gene expression level in CSC cluster. The following antibodies were used to represent the expression of the selected genes: anti-PARP1 (rabbit, 1:500, Abcam, ab32138), anti-PIGF (rabbit, 1:300, Proteintech, 10642-1-AP), anti-HDAC2 (rabbit, 1:500, Abcam, 32117), anti-FGFR3 (rabbit, 1:200, Abcam, ab137084), anti-BIRC5 (rabbit, 1:500, Abcam, ab76424), anti-PTTG1 (rabbit, 1:1000, Abcam, ab79546), anti-CENPF (rabbit, 1:500, Abcam, ab223847), anti-CDKN3 (rabbit, 1:500, Abcam, ab206314), anti-ATF3 (rabbit, 1:1000, Novusbio, nbp1-85816), anti-PDZK1 (mouse, 1:200, R&Dsystems, af4997), anti-VTN (rabbit, 1:300, Abcam, ab45139), anti-CXCL8 (mouse, 1:500, R&Dsystems, af-208-na)(Figure 4, Supplementary Figure 8).

### Gene set variation analysis (GSVA) and gene set enrichment analysis (GSEA)

Altogether 1329 canonical pathways in the website of molecular signature database (MSigDB, version 6.2) were provided by GSEABase package (version 1.44.0). Next, we applied GSVA method with default settings to assign pathway activity estimates for individual cells, as implemented in the GSVA package (version 1.30.0) 54. To quantify the differences in pathway activity between 16 clusters, we used a generalized linear model to contrast the enrichment scores for each cell. In addition, we applied the GSEA method 55 to demonstrate the significant differences of KEGG pathways between CSC and cancer 1-4 clusters.

### SCENIC analysis

The normalized expression matrix processed by Seurat package(version 2.3.4) was previously analyzed with SCENIC package based on 20-thousand motifs database for RcisTarget and GRNboost2 (SCENIC version 1.1.2.1, which corresponds to RcisTarget version 1.2.1 and AUCell version 1.4.1) 28, 56. Altogether 8774 genes passed the filtering (sum of expression >3 × 0.01× 10551 and detected in at least 1% of the cells). Next, GRNBoost2 from arboreto was used to infer co-expression modules and obtain potential regulons. RcisTarget and AUCell were employed to trim modules for targets and evaluate the activity of the regulatory network on all the cells respectively.

### Monocle analysis

The Monocle package (version 2.99.0) was used to plot trajectories to illustrate the behavioral similarity and transitions 57, 58. We used an expression matrix derived from Seurat to build a CellDataSet for Monocle pipeline, and partition the cells into supergroups after dimensionality reduction. SimplePPT method was applied in organizing supergroups into a tree-like trajectory. Plot cell trajectory module was used to plot the trajectory and color the cells by subcluster type.

### scRNA-seq and copy number estimation

Genome-wide relative copy number estimation of cancer cell and CSC was performed using InferCNV (version 0.8.2) 59. The count data matrix was delivered from Seurat. Gene symbols determining gene coordinates were obtained by querying Ensemble via BioMart and the corresponding genes were located on the chromosomes. The basic parameters of InferCNV were set by default (cutoff value = 0.1). The ngchm function of inferCNV package was used to generate an interactive heat maps for visualization of CNV data, which allows exploration of the detail variation of the copy number through all the chromosomes 60 on the website http://www.ngchm.net/Downloads/ngChmApp.html with Supplementary Plot 1.

### Cell-cell communication analysis

To analyze cell-to-cell interactions in the scRNA-seq data, we used CellPhoneDB method (cellphonedb.org) 61 to deduce the ligand-receptor interaction between two cell types. To determine the most relevant interactions between various cell types, we considered only the ligand-receptor complexes expressed in more than 10% cells. Then, we compared each cell cluster in pairs according to the default setting of CellPhoneDB, and calculated the number of important ligand-receptor complexes between each cluster (p<0.05), so as to estimate the communication between each cell cluster.

### RNA velocity

According to the previous reports 62, we used the spliced and unspliced transcript reads to calculate the RNA velocity. The spliced and unspliced reads were processed by R script velocyto.R with the Cell Ranger output. All the cells belonging to CSC and cancer cells were analyzed (n=10551). Velocity fields were projected onto the t-SNE plot generated by Seurat. Parameter n sight was 30 which determines the projection size of the velocity.

### Survival analysis

The IHC score and Kaplan-Meier survival curve were used to evaluate the relationship between gene (BIRC5, PTGG1, CENPF, CDKN3, ATF, PDZK1, VTN, CXCL8) expression and prognosis. First, ROC curve was used to distinguish the high and low expression cut off values of IHC scores (SPSS 24). Then, the survival status and time of patients were used to draw Kaplan-Meier curve (P < 0.01 indicated statistical difference).

### Statistical analysis

All single-cell sequencing data statistical analysis was performed in R (version 3.5.2) and Python (version 3.7.1). Wilcoxon Rank Sum test and Kruskal-Wallis rank sum test were applied for comparisons in two or more groups. Statistical significance was accepted for P < 0.05. In single cell RNA-seq data, we used Limma package to fit a generalized linear model, and Bayes moderated F-statistic to determine statistical significance.

## Supplementary Material

Supplementary figures.

Supplementary table 1.

Supplementary table 2.

Supplementary table 3.

Supplementary table 4.

Supplementary table 5.

Supplementary table 6.

Supplementary table 7.

Supplementary plot 1.
