# Supplementary material for: Identification of a novel cancer stem cell subpopulation that promotes progression of human fatal renal cell carcinoma by single-cell RNA-seq analysis
Source: Int J Biol Sci. 2020 Oct 17;16(16):3149–62. doi: 10.7150/ijbs.46645 (PMC7645996; doi:10.7150/ijbs.46645)
Supplement: Supplementary file 1 — Supplementary figures. [file ijbsv16p3149s1.pdf]

Supplementary Figure Legends

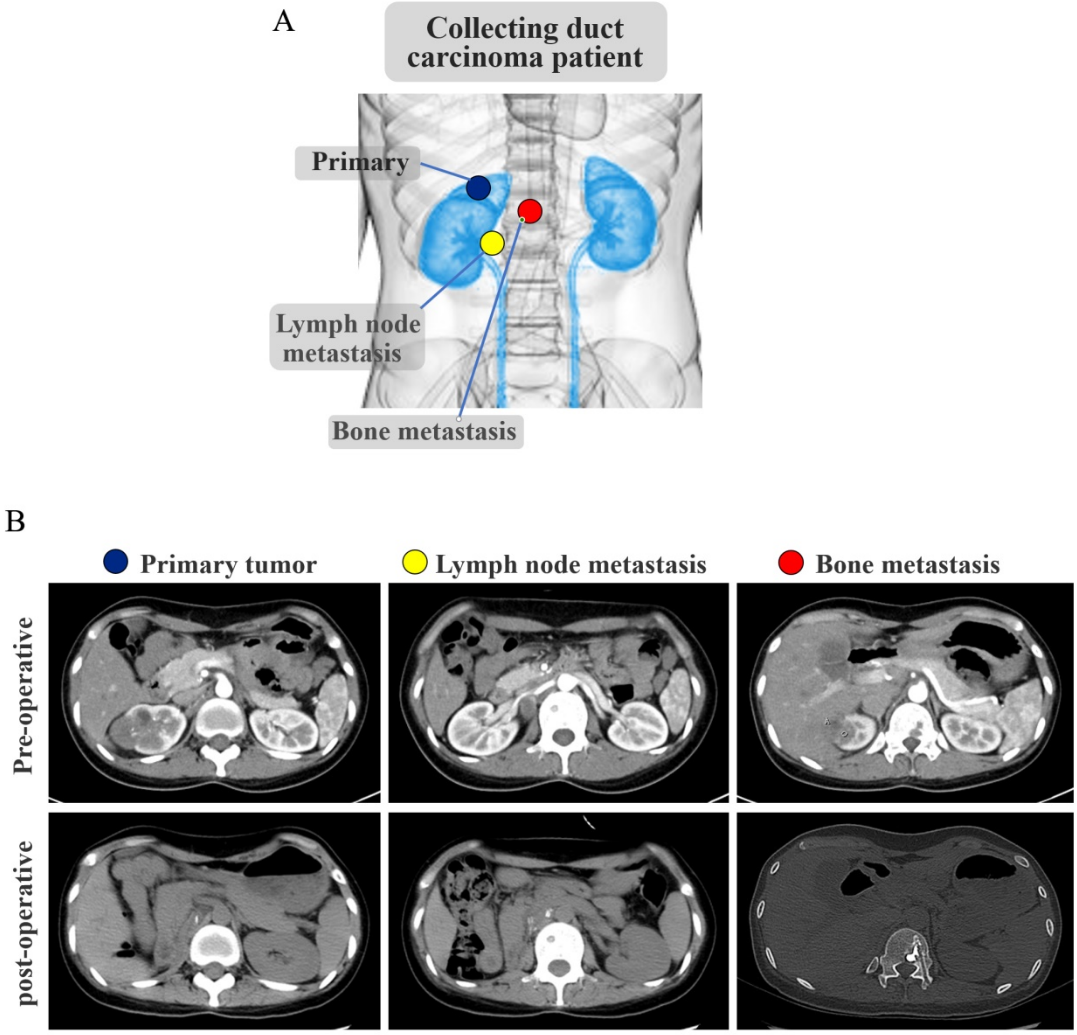

Supplementary Figure 1. Presentation of tumor site and imaging of the samples. A, Sampling site of the patient: All the samples originated from one patient simultaneously. The primary carcinoma and lymph node (LN) metastasis were obtained from the cytoreductive nephrectomy and the bone metastasis was obtained from the spinal biopsy. **B**, Representative preoperative and postoperative computed tomography (CT) of the patient undergoing cytoreductive nephrectomy and spinal biopsy. Contrast-enhanced CT was used for preoperative evaluation and plain CT was used for postoperative follow-up.

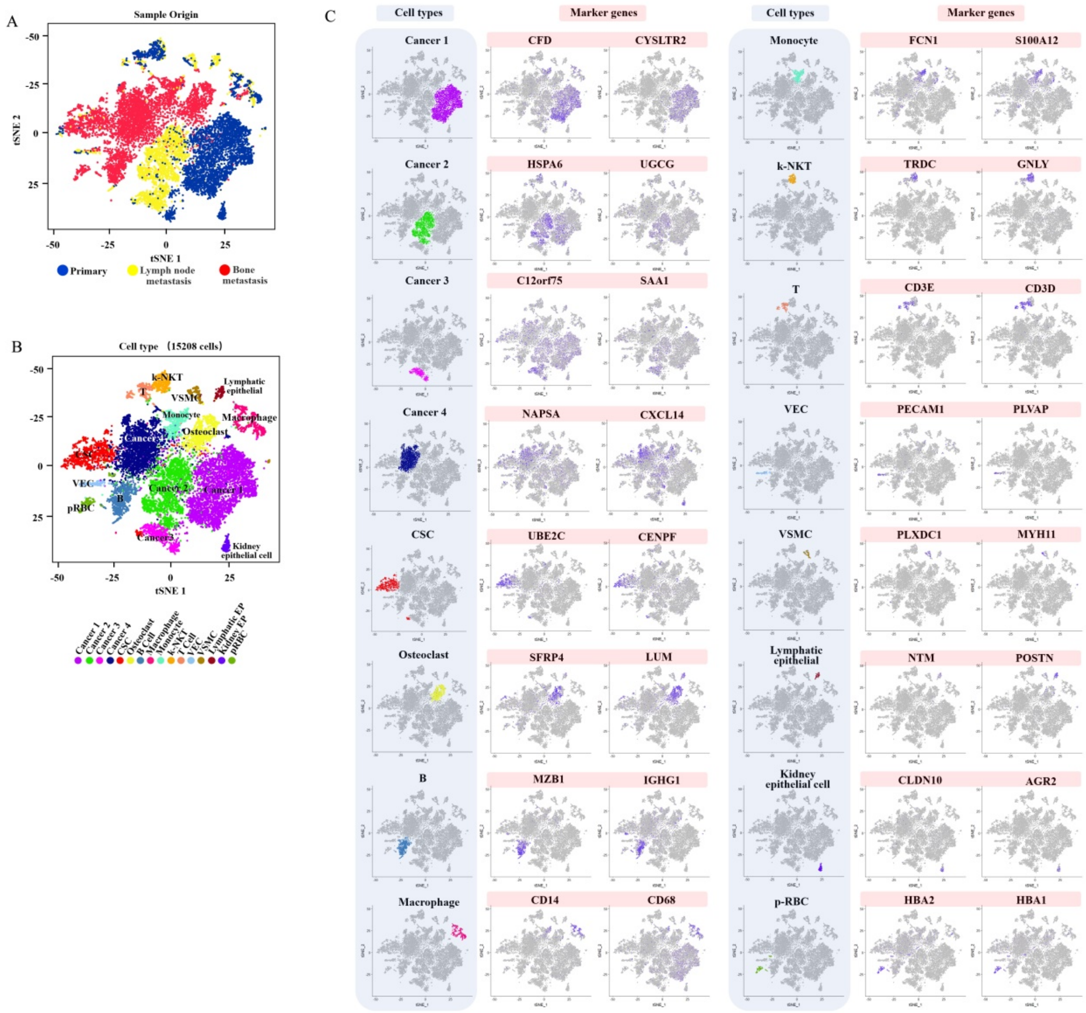

Supplementary Figure 2. Profile of t-SNE plot of single-cell RNA-seq data and the cell type classification. A, t-SNE plot of the 15208 cells shows the formation of primary tumor(n=4984), LN metastasis(n=3568) and bone metastasis(n=6656), each dot representing one single cell. **B**, t-SNE plot of the 15208 cells shows the formation of 16 clusters which were labelled by different colors. All the clusters are Cancer1~4, CSC, Osteoclast, B Cell, Macrophage, Monocyte, k-NKT, T cell, VEC, VSMC, Lymphatic EP, kidney EP and RBC. **C**, t-SNE plot shows the expression of specific marker genes identifying the 16 clusters. Markers for cancer 1 : genes encoding Complement Factor D(CFD), Cysteinyl Leukotriene Receptor 2(CYSLTR2); For Cancer 2 : genes encoding Heat Shock Protein Family A (Hsp70) Member 6(HSPA6), UDP-Glucose Ceramide Glucosyltransferase(UGCG); For Cancer 3 : genes encoding Chromosome 12 Open Reading Frame 75(C12orf75), Serum Amyloid

A1(SAA1); For Cancer 4 : genes encoding Napsin Aspartic Peptidase(NAPSA), C-X-C Motif Chemokine Ligand 14(CXCL14); For CSC : genes encoding C-X-C Motif Chemokine Ligand 14(UBE2C), Centromere Protein F(CENPF); For Osteoclast : genes encoding Secreted Frizzled Related Protein 4(SFRP4), Lumican(LUM); For B cells : genes encoding Marginal Zone B And B1 Cell Specific Protein(MZB1), Immunoglobulin Heavy Constant Gamma 1(IGHG1); For Macrophage : genes encoding CD14 Molecule(CD14), CD14 Molecule(CD68); For Monocyte : genes encoding Ficolin 1(FCN1), S100 Calcium Binding Protein A12(S100A12); For k-NKT : genes encoding T Cell Receptor Delta Constant(TRDC), Granulysin(GNLY); For T cells : genes encoding CD3e Molecule(CD3E), CD3d Molecule(CD3D); For VEC : genes encoding Platelet And Endothelial Cell Adhesion Molecule 1(PECAM1), Plasmalemma Vesicle Associated Protein(PLVAP); For VSMC : genes encoding Plexin Domain Containing 1(PLXDC1), Myosin Heavy Chain 11(MYH11); For Lymphatic epithelial : genes encoding Neurotrimin(NTM), Periostin(POSTN); For kidney epithelial cells : genes encoding Claudin 10(CLDN10), Anterior Gradient 2, Protein Disulphide Isomerase Family (AGR2); For RBC : genes encoding Hemoglobin Subunit Alpha 2(HBA2), Hemoglobin Subunit Alpha 1(HBA1).

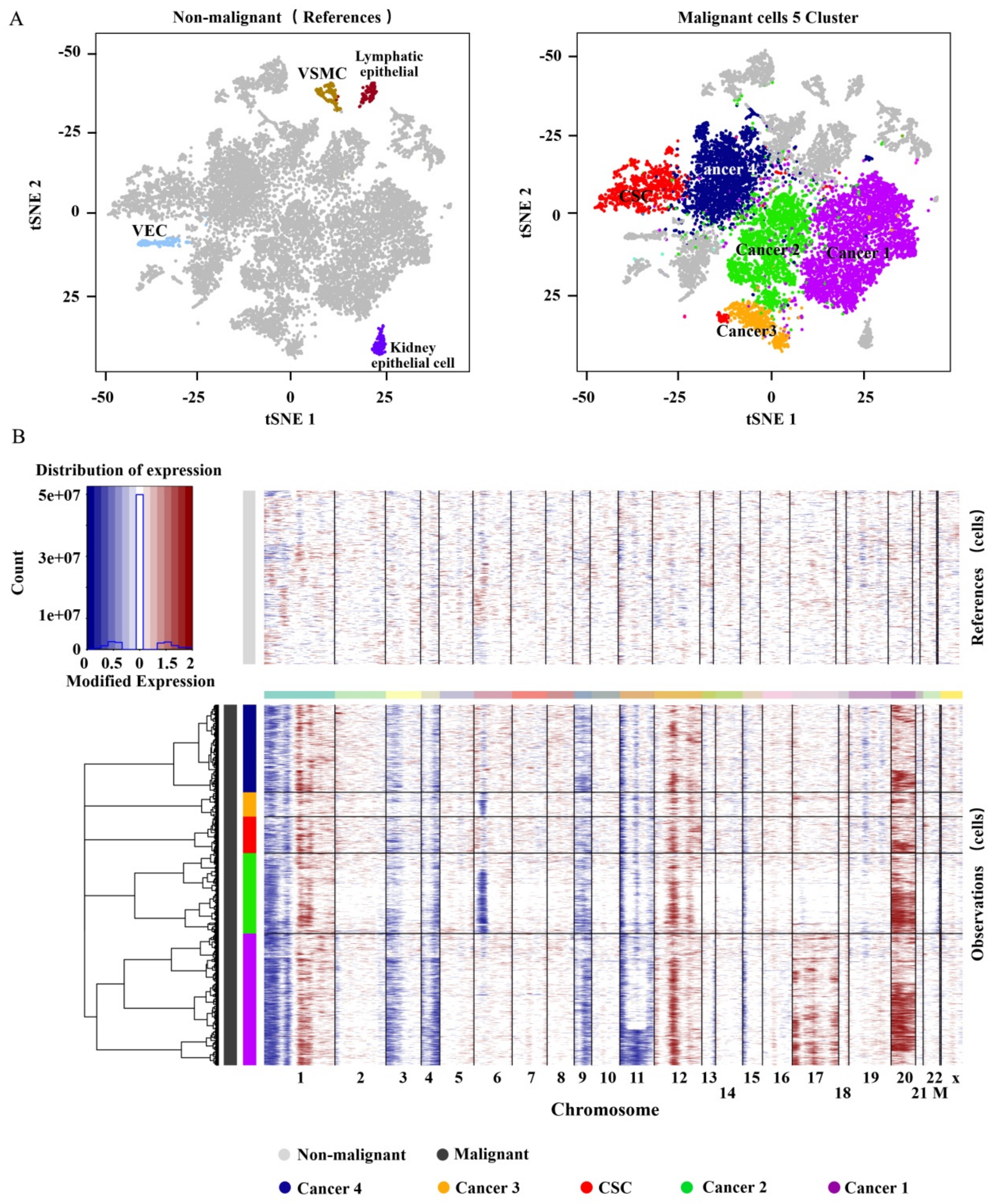

Supplementary Figure 3. Chromosomal transcriptional heterogeneity between malignant and nonmalignant cells. A, t-SNE plot of four nonmalignant cell clusters(VSMC, VEC, lymphatic epithelial and kidney epithelial cell) and five malignant cell clusters(cancer1~4 and CSC). **B**, Chromosomal heatmap of inferred large-scale Copy Number Variations (inferCNV, see methods) shows the copy number variation difference between malignant and nonmalignant cells. Copy number changes were inferred in 23 chromosomes(columns) for five cluster cells(rows) .

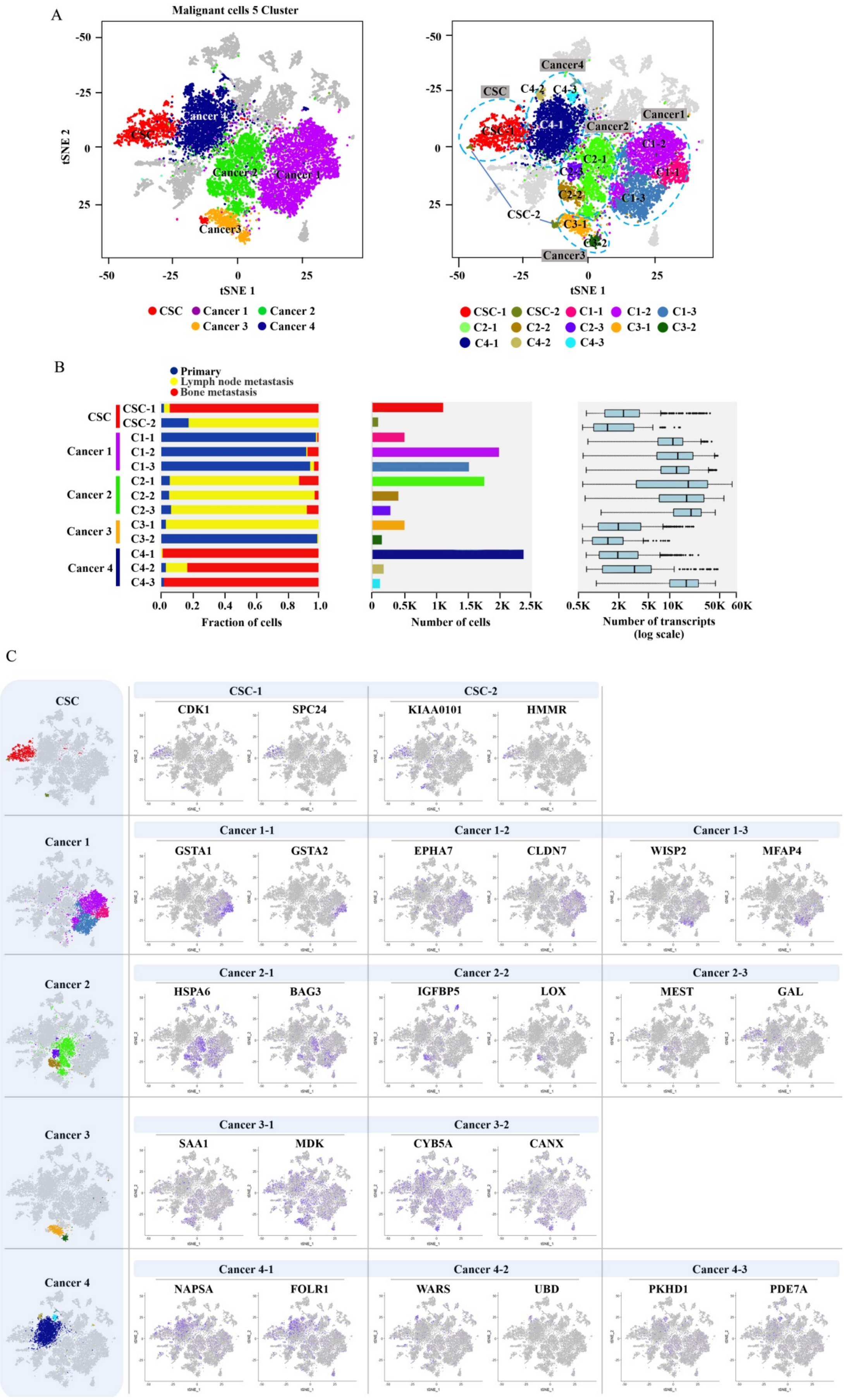
A
Malignant cells 5 Cluster
tSNE 1
tSNE 2
CSC
Cancer 1
Cancer 2
Cancer 3
Cancer 4
Cancer1
Cancer2
Cancer3
Cancer4
CSC-1
CSC-2
C1-1
C1-2
C1-3
C2-1
C2-2
C2-3
C3-1
C3-2
C4-1
C4-2
C4-3
B
Primary
Lymph node metastasis
Bone metastasis
Fraction of cells
Number of cells
Number of transcripts (log scale)
C
CDK1
SPC24
KIAA0101
HMMR
Cancer 1-1
GSTA1
GSTA2
Cancer 1-2
EPHA7
CLDN7
Cancer 1-3
WISP2
MFAP4
Cancer 2-1
HSPA6
BAG3
Cancer 2-2
IGFBP5
LOX
Cancer 2-3
MEST
GAL
Cancer 3-1
SAA1
MDK
Cancer 3-2
CYB5A
CANX
Cancer 4-1
NAPSA
FOLR1
Cancer 4-2
WARS
UBD
Cancer 4-3
PKHD1
PDE7A

Supplementary Figure 4. Profile of cancer and CSC subclusters. A, Subclusters of cancer and CSC identified by distinctively expressed marker genes labelled with different colors. The CSC cluster was further divided to CSC-1 and CSC-2, meanwhile the cancer clusters were divided to C1-1, C1-2, C1-3, C2-1, C2-2, C2-3, C3-1, C3-2, C4-1, C4-2 and C4-3. **B**, The bar plot (from left to right) depicts the fractions of 13 subclusters classified in cancer and CSC cluster and the corresponding number of cells and transcripts. **C**, The expression of specific marker genes identifies the subclusters of cancer and CSC clusters. For CSC-1: genes encoding Cyclin Dependent Kinase 1(CDK1), SPC24 Component Of NDC80 Kinetochore Complex(SPC24); For CSC-2 : genes encoding PCNA Clamp Associated Factor(KIAA0101), Hyaluronan Mediated Motility Receptor(HMMR); For Cancer 1-1: genes encoding Glutathione S-Transferase Alpha 1(GSTA1), Glutathione S-Transferase Alpha 2(GSTA2); For Cancer 1-2 : genes encoding EPH Receptor A7(EPHA7), Claudin 7(CLDN7); For Cancer 1-3 : genes encoding Cellular Communication Network Factor 5(WISP2), Microfibril Associated Protein 4(MFAP4); For Cancer 2-1 : genes encoding Heat Shock Protein Family A (Hsp70) Member 6(HSPA6), BCL2 Associated Athanogene 3(BAG3); For Cancer 2-2: genes encoding Insulin Like Growth Factor Binding Protein 5(IGFBP5), Lysyl Oxidase(LOX); For Cancer 2-3 : genes encoding Mesoderm Specific Transcript(MEST), Galanin And GMAP Prepropeptide(GAL); For Cancer 3-1 : genes encoding Serum Amyloid A1(SAA1), Midkine(MDK); For Cancer 3-2 : genes encoding Cytochrome B5 Type A(CYB5A), Calnexin(CANX); For Cancer 4-1 : genes encoding Calnexin(NAPSA), Folate Receptor 1(FOLR1); For Cancer 4-2 : genes encoding Tryptophanyl-TRNA Synthetase(WARS), Ubiquitin D(UBD); For Cancer 4-3 : genes encoding PKHD1 Ciliary IPT Domain Containing Fibrocystin/Polyductin(PKHD1), Phosphodiesterase 7A(PDE7A).D, The violin plot shows the co-expression of marker genes from CDRCC and CCRCC CSCs. ENG,CXCR4 and ALDH1A1 are the markers of CCRCC CSCs and the MKI67,

TOP2A,CENPF,CDK1,UBE2C and KIAA0101 are the markers of CDRCC CSCs.

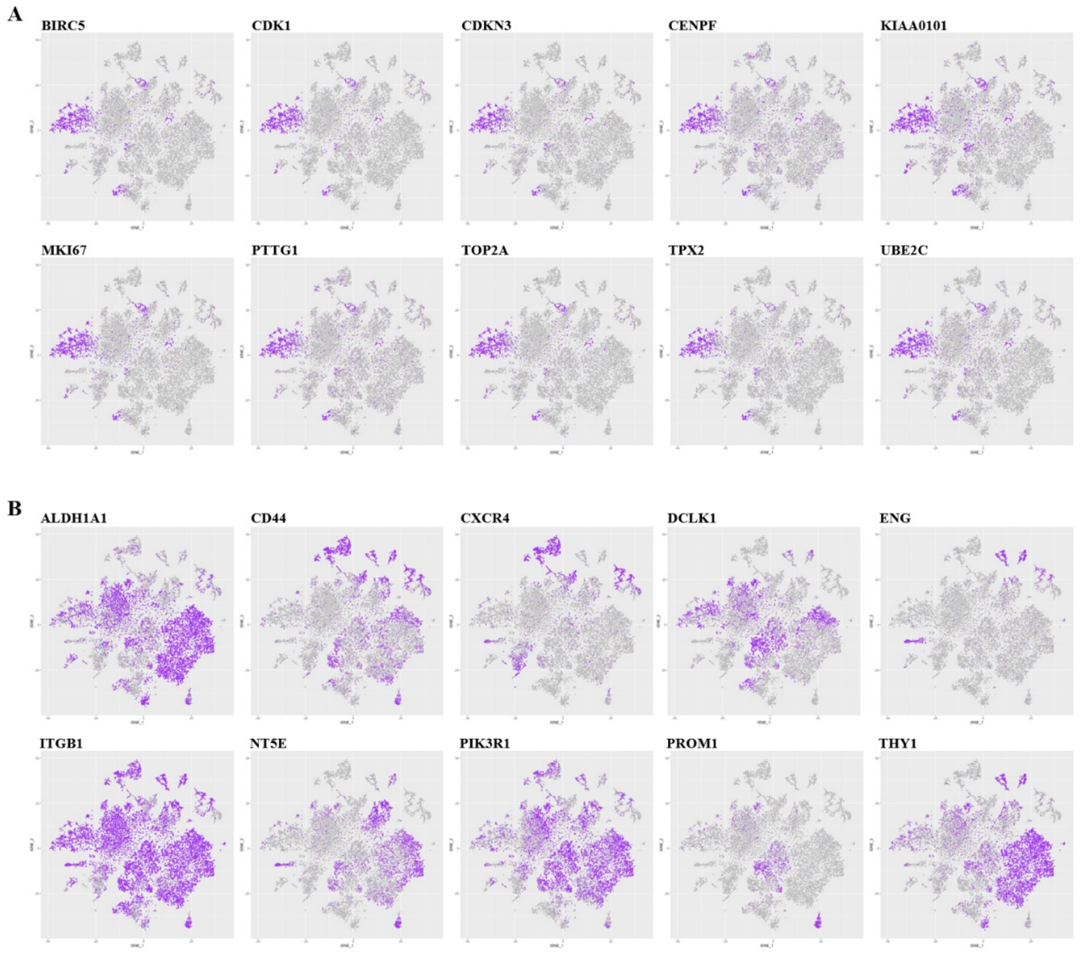

Supplementary Figure 5. Expression of CSC markers in CDRCC subclusters. A, Featureplot shows the expression of specific marker genes identifies the subclusters of CDRCC CSC clusters from our research. **B**, Featureplot shows the expression of specific marker genes of CCRCC CSC in the literature in CDRCC subclusters.

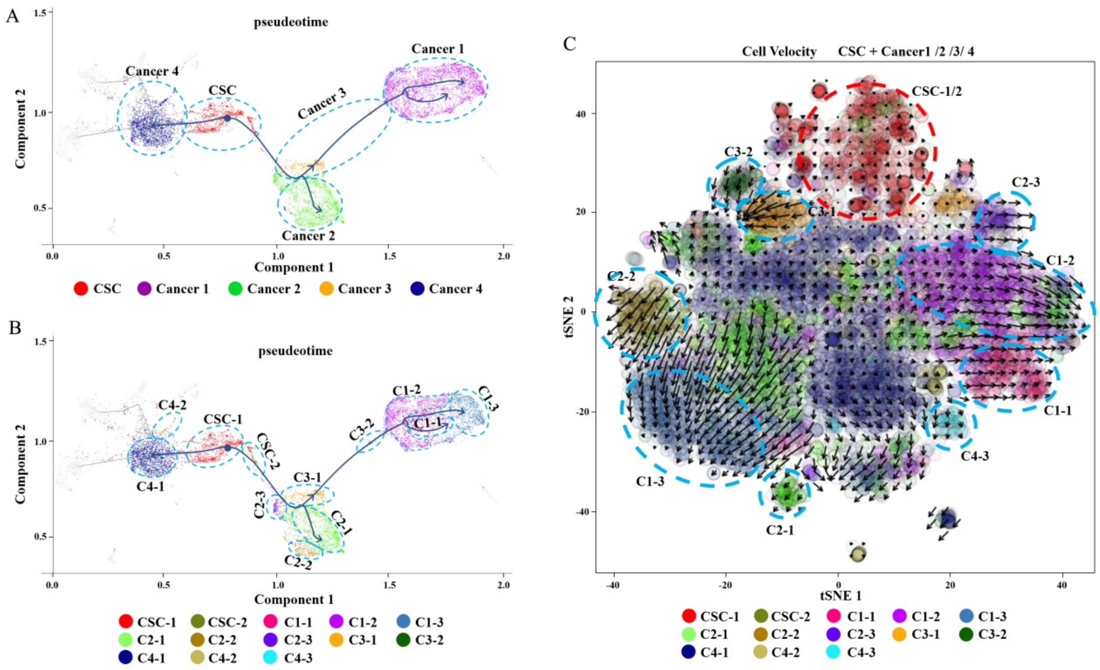

Supplementary Figure 6. RNA velocity elucidated the differentiation kinetics of CSC to cancer subclusters. A,**B**, Monocle pseudeotime trajectory predicting the potential evolution of CSC to cancer clusters, the arrows represent the direction of cell differentiation. **C**, Velocity field showing in the t-SNE plot among CSC and Cancer subclusters. The directions of arrows represent the future state of the cells.

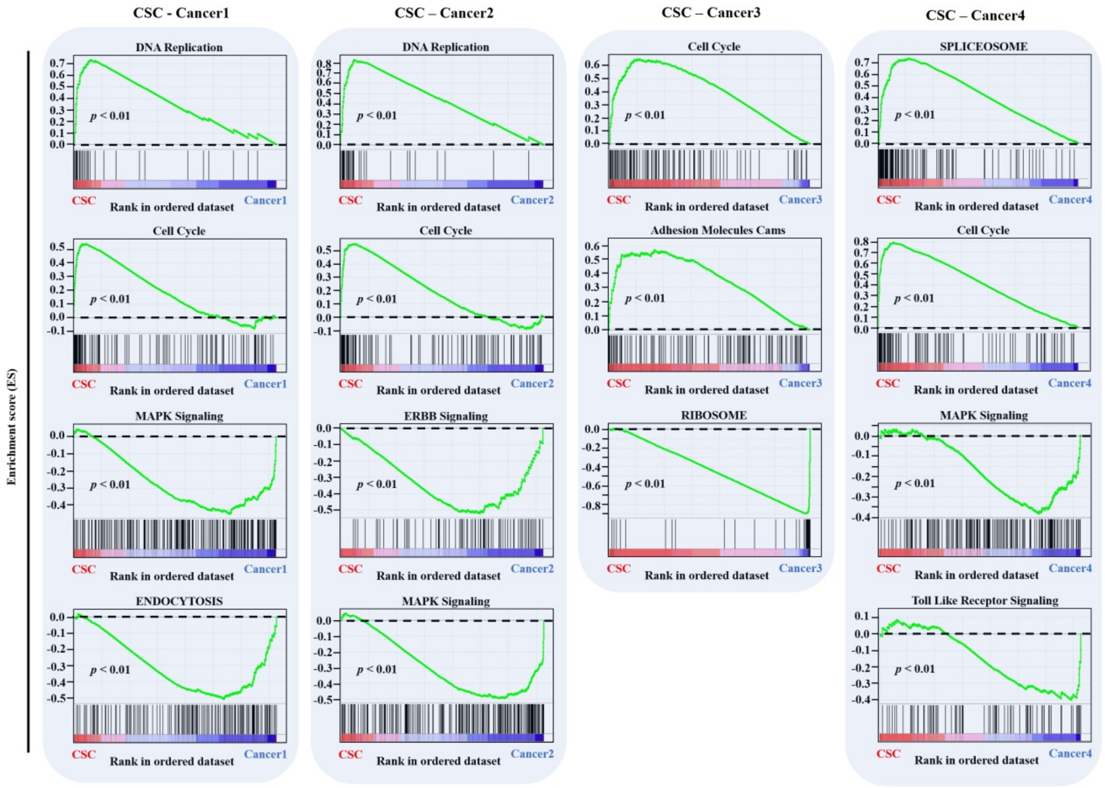

Supplementary Figure 7. GSEA plot showing the enrichment of the signal pathway between CSC and Cancer clusters(Cancer1~4). The signal pathway of DNA replication, Cell cycle, SPLICEOSOME and Adhesion Molecules Cams were enriched in the CSC cluster, nevertheless signal pathway, such as MAPK, RIBOSOME, Toll Like Receptor, ENDOCYTOSIS and ERBB signal pathway were enriched in Cancer clusters(p<0.01).

A

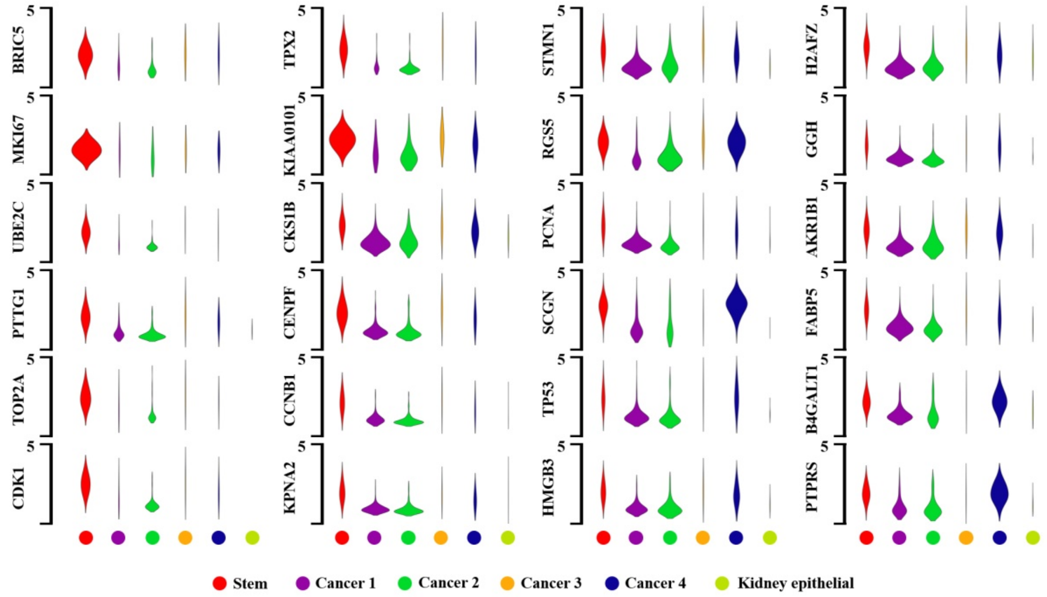

B

Gene symbol	logFC	*P*_val	References
AKR1B1	0.933683265	2.31E-89	[1,2]
ARL6IP1	0.933812736	1.81E-64	[3]
B4GALT1	0.30131245	9.65E-24	[4]
BIRC5	0.902472745	0	[5-7]
CBX6	0.360552926	4.97E-19	[8]
CCNB1	1.162033583	1.69E-181	[9,10]
CDK1	0.990415031	0	[11-14]
CENPF	1.5239161	0	[15]
CKS1B	1.115315342	2.06E-134	[16]
EIF5A	0.748184657	4.67E-87	[17]
FABP5	0.650676917	7.86E-53	[18]
H2AFZ	1.101764377	2.59E-146	[19-23]
HIST1H4C	1.268127353	4.26E-148	[3]
HMGA1	0.593608864	7.60E-22	[24-26]
HMGB2	0.819137337	1.53E-45	[27-29]
HMGB3	0.77358788	3.81E-70	[30-35]
HNRNPH1	0.657982368	8.01E-91	[36]
IGFBP2	0.316352512	7.67E-13	[37]
KIAA0101	1.328395823	0	[38-40]
KPNA2	1.209587597	2.84E-150	[41]
MKI67	0.943840275	0	[42]
MMP7	0.506896752	4.91E-88	[43]
NUCKS1	0.800250638	2.84E-145	[44]
PCNA	1.011322586	5.33E-72	[45-47]
PTPRS	0.447536095	9.49E-42	[48]
PTTG1	1.387605159	0	[49-53]
RAN	0.888485148	1.42E-193	[54]
RANBP1	0.881145057	3.31E-84	[55,56]
RGS5	0.725446647	4.83E-129	[57]
SET	0.679416442	1.32E-70	[58]
STMN1	1.025748327	4.52E-126	[59-61]
TM4SF4	0.464659732	9.37E-13	[62]
TOP2A	1.135011066	0	[63,64]
TP53	0.455415421	7.26E-21	[65-68]
TPX2	1.044481871	0	[69]
TUBA1B	1.391173937	0	[70]
TUBB	1.119028521	1.25E-244	[36]
UBE2C	1.889964808	0	[71,72]

Supplementary Figure 8. The expression difference of CSC related genes. **A**,Violin plot shows the expression level of the CSC related genes in CSC, Cancer and nonmalignant clusters. **B**, The CSC-related genes reported in the previous literature show significant differences in our single-cell data(*P*<0.01)(Supplementary Table 7).

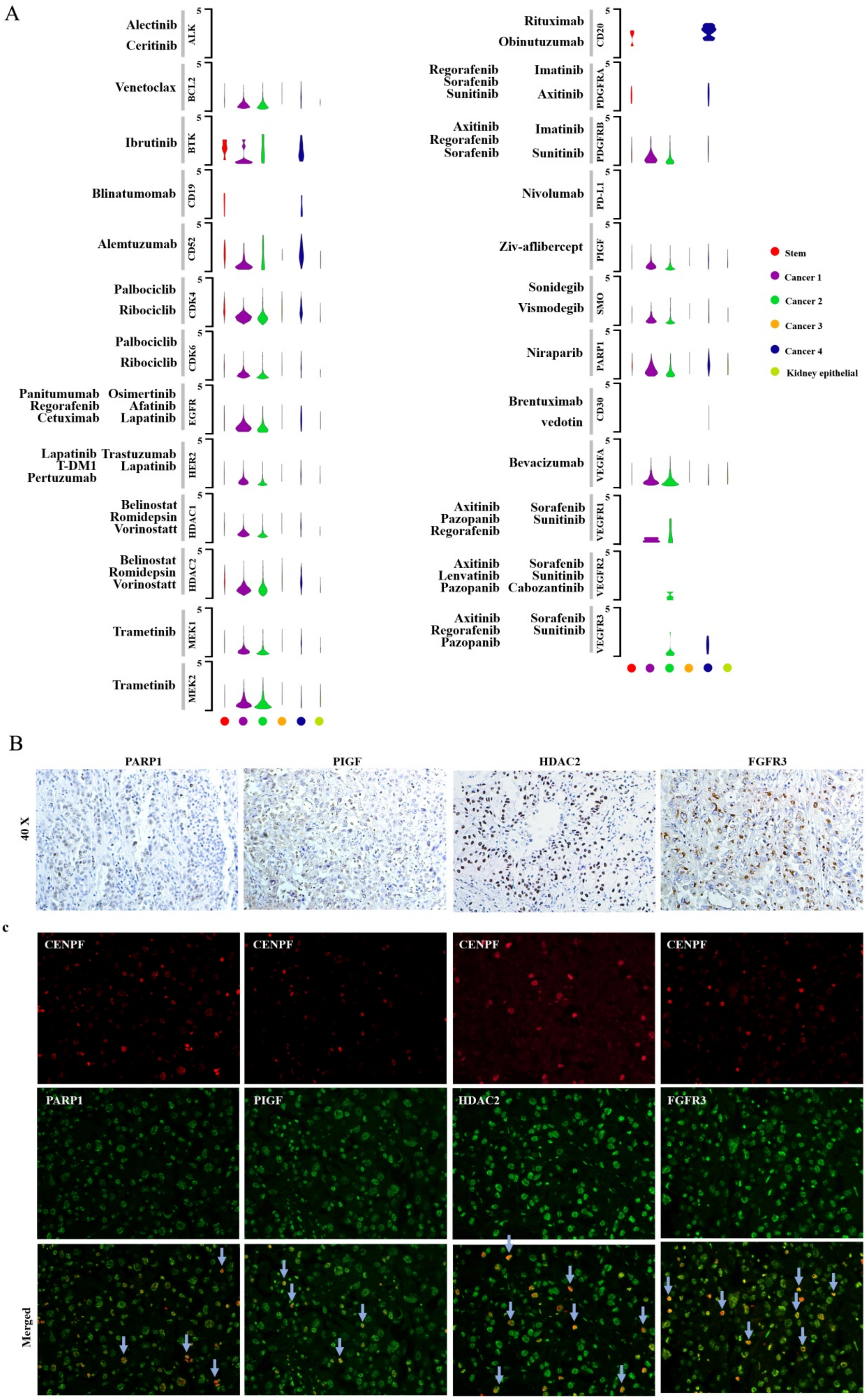

Supplementary Figure 9. Potential therapeutic responses for human

CDRCC to molecular targeted therapy. A, Violin plot shows the expression of target genes for the common targeted drugs in CSC, Cancer and nonmalignant clusters. **B**, Immunohistochemical images show the expression of targeted drug associated genes PARP1, PIGF, HDAC2, FGFR3 in the samples of CDRCC using the 40× objective. **C**, Double immunofluorescence staining was performed with the CSC related gene CENPF and the targeted drug associated genes as described in (**B**), and the fluorescence of the two dyes overlapped.
